# Evaluating Retrieval-Augmented Large Language Models on Anesthesiology Board-Style Questions: Benchmark Study

**DOI:** 10.2196/97902

**Published:** 2026-08-11

**Authors:** Nguyen Quang Phuong, Shanq-Jang Ruan, Pei-Fu Chen

**Affiliations:** 1 Department of Electronic and Computer Engineering, National Taiwan University of Science and Technology, Taipei, Taiwan; 2 Department of Anesthesia, University of Iowa, 200 Hawkins Drive, 6 JCP, Iowa City, IA, 52242, United States, 1 319-356-2502

**Keywords:** retrieval-augmented generation, anesthesiology board examination, large language model, reasoning model, embedding model

## Abstract

**Background:**

Open-source, mid-scale large language models (LLMs) have emerged as scalable, privacy-preserving alternatives to ultra-large foundation models (eg, GPT-4) in health care systems. Techniques such as retrieval-augmented generation (RAG) enable sub-100-billion-parameter models to address highly specialized medical domains such as anesthesiology. However, studies evaluating RAG architectures on complex medical examinations remain scarce, highlighting the need for rigorous benchmarking to bridge the gap between raw parametric knowledge and clinically relevant application.

**Objective:**

This study aimed to systematically evaluate RAG pipelines for answering anesthesiology board-style questions, quantify the effects of key design choices including hyperparameter settings, embedding models, source complexity, and chunking strategies, and compare the performance of reasoning-oriented models with that of conventional LLMs.

**Methods:**

We conducted large-scale benchmarking using American Board of Anesthesiology-style multiple-choice questions to compare multiple RAG-enabled configurations with matched standalone LLM baselines. Configurations were first optimized on a 46-item diagnostic set and then validated on a 350-item corpus. Additional experiments on three 100-question subsets derived from the 350-item corpus were used to assess the effects of source selection, source complexity, information density, and chunking strategy on answer accuracy. Models including Llama-3-8B-Instruct, Llama-3.1-8B-Instruct, Llama-3.2-3B-Instruct, Llama-3.3-70B-Instruct, Qwen2.5-7B and Qwen2.5-72B, and Qwen3-8B and Qwen3-32B reasoning models were evaluated under this framework. Self-reflective RAG (self-RAG) with adaptive retrieval techniques was also implemented and evaluated. Cochran Q and McNemar tests were used to assess performance differences across configurations and model pairs.

**Results:**

The RAG framework increased the number of correct answers. System stability peaked under highly deterministic sampling configurations (temperature=0.1, top-p [nucleus sampling]=0.1). High-capacity general-text embeddings and applying context-preserving semantic chunking further improved accuracy. Standard RAG provided only modest gains over nonaugmented baselines, improving accuracy from 50.29% to 56.57%, and self-RAG yielded similarly limited gains of up to 4.85 percentage points. Overall, the Qwen family outperformed the Llama series. The 32-billion-parameter reasoning model Qwen-3-32B achieved an 89% correct ratio under complex distractor-heavy retrieval conditions and up to 96% with direct context, significantly outperforming the much larger 72-billion-parameter conventional model Qwen-2.5-72B-Instruct (84%). Smaller reasoning models also showed greater robustness to noise or suboptimal retrieved documents than larger conventional LLMs. Within the Llama family, increasing parameter size to 70 billion did not produce proportional performance gains on this benchmark.

**Conclusions:**

RAG-based LLM systems improved performance on anesthesiology board-style questions, but gains depended strongly on retrieval design. Careful optimization of retrieval settings, embeddings, and chunking strategies improved robustness and answer accuracy. Reasoning-oriented models demonstrated that multistep reasoning can, in some settings, compensate for larger parameter scale. These findings provide a methodological foundation for developing locally deployable LLM systems for anesthesiology education within structured examination settings.

## Introduction

Large language models (LLMs), ranging from millions to trillions of parameters, have evolved from general-purpose chatbots to tools under active study across health care delivery, research, and education, with early evidence synthesized across multiple domains [1]. Reviews emphasize both the promise of LLMs to support clinical workflow and answer medical examination questions, as well as the need to mitigate risks such as bias and hallucinations [2]. Methodological guidance calls for transparent, task-representative benchmarking, standardized reporting, and reproducible evaluation to determine clinical or educational utility [3]. A meta-analysis of ChatGPT (OpenAI) versions across medical licensing examinations found that newer techniques tend to achieve higher pass rates, yet performance varied by different question types [4].

A comprehensive review of 16 LLMs across 198 medical exams reported that GPT-4 outperformed many peers, passing 131 of 262 exam sets (50%) [5]. On United States Medical Licensing Examination (USMLE) question sets (AMBOSS Step 1/2 and NBME Free Step 1/2), ChatGPT achieved 42%‐64.4% accuracy and exceeded the 60% passing threshold on NBME-Free-Step1 [6]. In a separate analysis, ChatGPT approached or met the passing threshold across USMLE Steps 1, 2 CK, and 3, indicating potential for clinical education [7]. More recently, GPT-4 Omni had outscored earlier models across USMLE disciplines and clinical skills but still showed content-area variability [8]. Collectively, these findings indicate that even state-of-the-art models can experience performance declines as item difficulty increases, underscoring the need to validate robustness across difficulty strata and exam blueprints.

LLMs demonstrated strong performance on general medical exams, with accuracy very close to the pass threshold, which has motivated experiments in the discipline-specific medical domain, such as the American Board of Anesthesiology (ABA) exam. In the ABA-style exam, an early study found that GPT-4 outperformed GPT-3.5 and Bard on review-book questions, yet still fell short of the 70% passing threshold, offering explanations that were not consistently aligned with medical consensus. This highlights the need for domain-aligned methods and evaluation [9]. In the Japanese Society of Anesthesiologists’ written exam, GPT-4 (~50%) outperformed GPT-3.5 but still fell below the passing cutoffs, with specialty-specific and item-format constraints limiting accuracy [10]. A human-benchmarked study on the Chilean anesthesiology certification exam revealed that reasoning models led (GPT-o1, 88.7%), with accuracy declining as items became more challenging; most errors occurred in application or understanding tasks and involved flawed reasoning or incorrect application of knowledge [11]. For the ABA exam specifically, GPT-4 was reported to pass BASIC and ADVANCED sections with scores of 78% and 80%, but performance still varied by topic area [12]. In parallel, a letter from the Royal College of Anesthetists, reporting 27 questions, noted that GPT4 achieved a significantly higher average score of 67% correct than GPT3.5’s 43%, and flagged concerns about bias, transparency, and the explainability of generated answers [13]. While another study found LLMs exceeding the 70% threshold on ABA BASIC-style sets, these analyses, which use publicly available BASIC items and assumptions about item reuse, complicate inferences about actual ABA readiness and its effects on physicians’ training and assessment [14,15].

Augmenting models with domain-specific sources via retrieval-augmented generation (RAG) improved internal medicine board performance for GPT-3.5/4 by approximately 4.5%‐7.5% when accessed via the application programming interface. However, the interface choice itself affected scores, with application programming interface results 3.2%‐5.3% lower than chatbot results [16]. A multilingual benchmark with gold rationales further showed that, even with state-of-the-art RAG, English accuracy plateaued around 75% and was about 10 percentage points lower in other languages, highlighting persistent challenges in retrieving the proper evidence and in evaluating reasoning quality [17]. RAG can improve the accuracy of smaller models and narrow the gap to larger models; however, current evidence does not show consistent parity across medical exams [16,17]. Model sizes were not consistently reported, and neither study establishes size-based parity [16,17]. Smaller models can also support on-premises or locally hosted deployment, which may better align with privacy-preserving requirements and regulatory expectations in clinical settings [18].

Together, these observations define the gap: comprehensive, reliability-focused studies in anesthesiology that directly test when and how advanced RAG, through source selection, source complexity, and chunking, improves performance on ABA-style questions remain scarce. Accordingly, we evaluate LLM performance on an extensive ABA-style question bank, quantify the incremental gains of RAG over bare generation, and systematically vary source selection and complexity while contrasting naive versus semantic chunking with exam-aligned materials [19]. We also evaluate recently released reasoning models that integrate a unified, multistep reasoning approach to quantify their incremental value over conventional models, thereby strengthening the applicability and generalizability of our study [20].

This study makes 3 main contributions. First, we provide a systematic procedure for tuning and optimizing retrieval hyperparameters, as well as for selecting and implementing chunking strategies in RAG-enabled systems for medical education, with a focus on anesthesiology. Second, we conduct a comprehensive benchmark on an ABA-style question bank, evaluating multiple RAG configurations under standardized prompts and across graded retrieval-complexity tiers (bare, direct, combined easy, medium, and hard). Third, we demonstrate the incremental value of reasoning models over conventional language models on the same benchmark, quantifying their added benefit for medical examination question answering.

Accordingly, the aim of this study is to systematically benchmark RAG pipelines for answering anesthesiology board-style multiple-choice questions. Specifically, we evaluate how key design choices, including hyperparameter settings, embedding models, retrieval strategies, and chunking approaches, influence model performance. In addition, we compare reasoning-oriented models with conventional LLMs under varying retrieval complexity conditions. This study is positioned as a controlled benchmarking evaluation rather than a clinical validation, with the goal of providing methodological insights into optimizing RAG systems for medical education tasks.

## Methods

### Ethical Considerations

This study did not involve human participants, patient data, protected health information, biological specimens, or animal subjects. The study evaluated computational models using publicly available anesthesiology board-style educational materials. Therefore, institutional review board approval and informed consent were not required in accordance with institutional and applicable regulations. No personally identifiable information was collected, analyzed, or reported.

### Study Design

**Figure 1.**
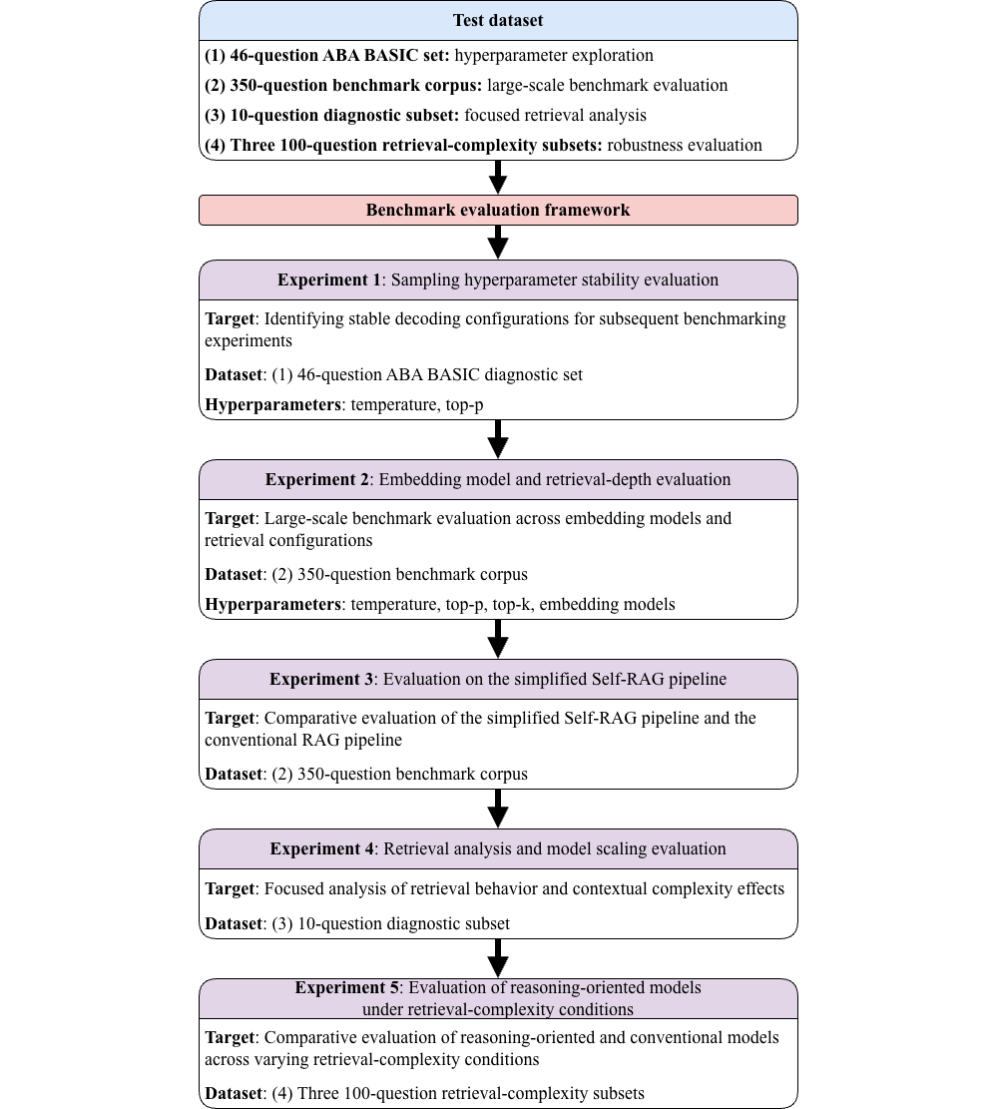
Overview of the experimental framework and benchmark datasets used throughout the study. The flowchart summarizes the purpose of each experiment, the associated datasets, and the progression from diagnostic hyperparameter exploration to large-scale retrieval and reasoning-oriented benchmarking experiments. ABA: American Board of Anesthesiology; RAG: retrieval-augmented generation.

To identify optimal configurations and establish a comprehensive benchmark, we conducted comparative experiments between RAG pipeline configurations and their nonaugmented baseline counterparts. To ensure robust evaluation, the study implemented and tested a diverse range of retrieval techniques and current model advancements. Figure 1 summarizes the overall experimental framework, benchmark datasets, and the relationship between the 5 experiments.

### Experiment 1: Searching for an Optimal Creativity Control

#### RAG Pipeline Architecture

**Figure 2.**
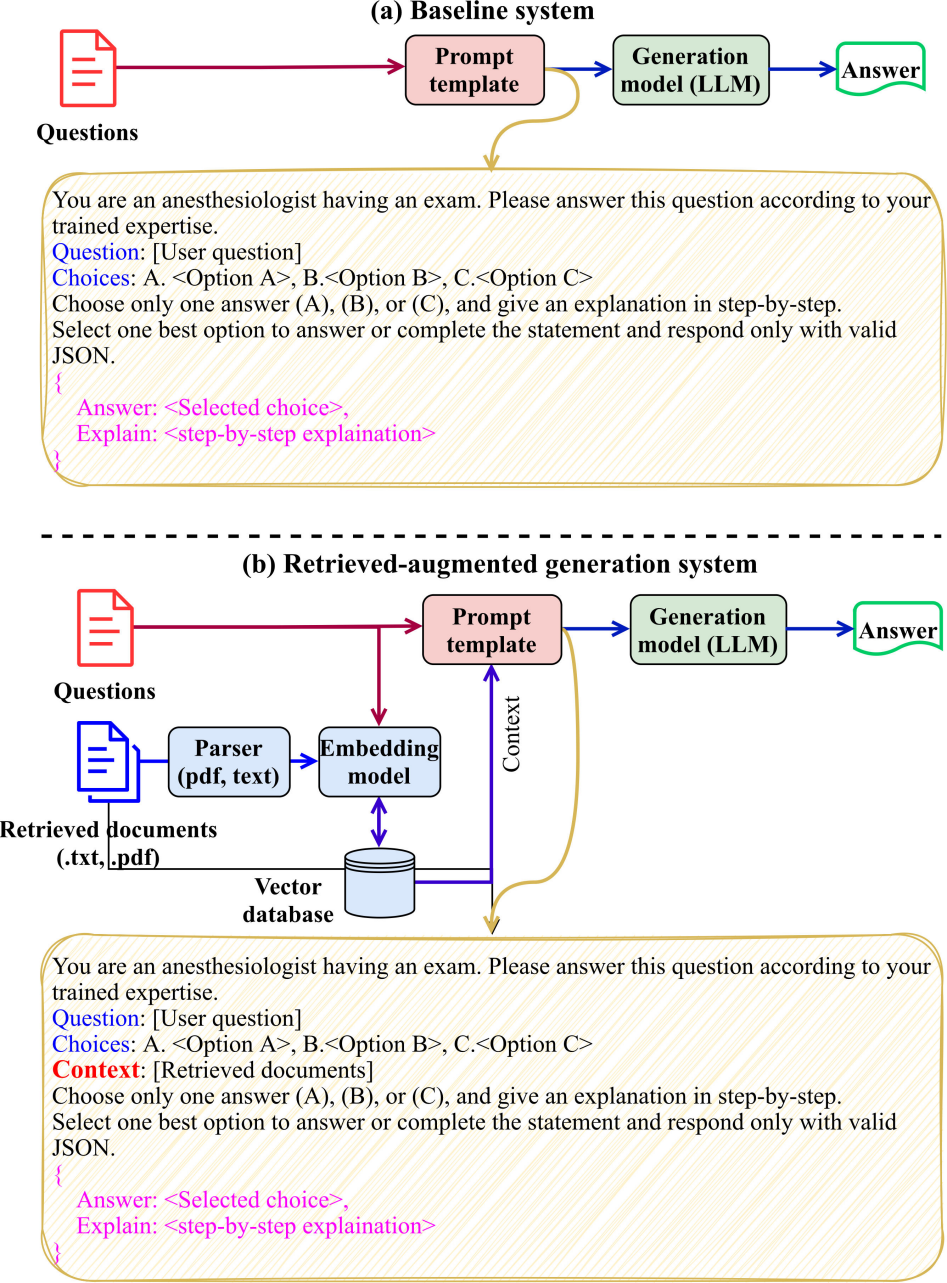
Schematic overview of the experimental system architectures used in this anesthesiology board-style question-answering benchmark study. LLM: large language model.

The RAG architecture, illustrated in Figure 2B, comprised five components: document parsing, embedding generation, vector storage, prompt engineering, and response generation. The workflow began with a parser module that segments the source documents into discrete semantic chunks. Subsequently, the embedding model transformed these segments into high-dimensional vector representations, which were indexed in a vector database to facilitate efficient semantic similarity search [21]. While higher vector dimensions correlate with increased text expressiveness, they also incur higher computational costs; therefore, our model selection balanced performance with efficiency. Relevant context is subsequently retrieved and integrated into the prompt template to guide the generation model.

During the retrieval stage, input queries (exam questions) were projected into the vector space, and relevant document chunks were retrieved based on cosine similarity scores. These retrieved contexts were integrated into a structured prompt template and forwarded to the generation model. Generated answers were evaluated against the ground truth to calculate accuracy metrics (ie, the percentage of correct answers). For comparison, a baseline evaluation was conducted in a standalone setting (Figure 2A), relying solely on the LLM’s internal parameters to generate responses.

#### Hyperparameter Optimization and System Configuration

**Figure 3.**
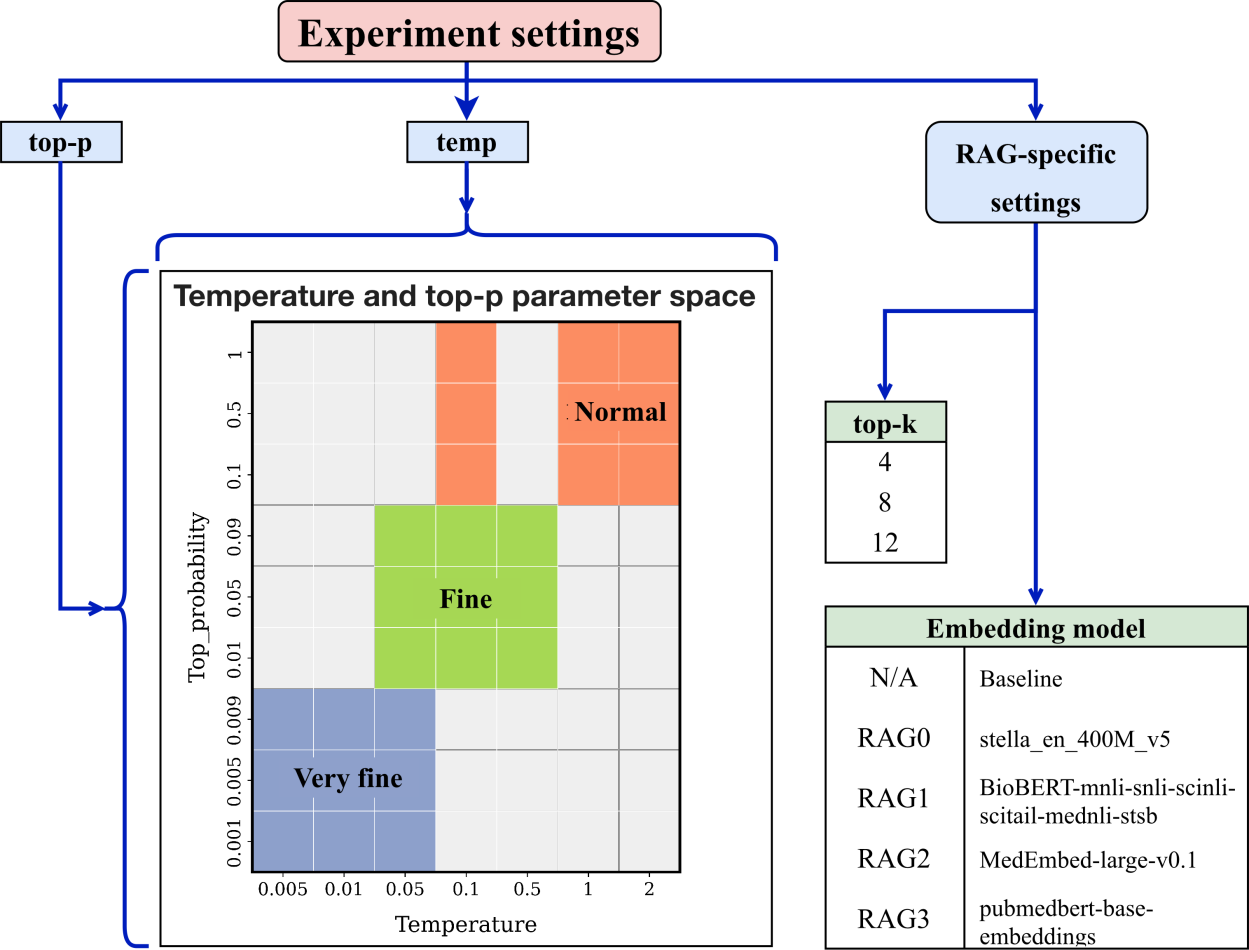
Configuration of experimental hyperparameters across the 46-question American Board of Anesthesiology-style validation subset. The generation model’s performance was evaluated across a parameter space defined by varying temperature and top-p values, and the results were clustered into three stability zones (“Normal,” “Fine,” and “Very Fine”). For the retrieval-augmented generation pipeline, distinct retrieval settings were tested, including three top-k depths (4, 8, 12) and four distinct embedding models (stella-en_400M_v5, BioBERT-mnli-snli-scinli-scitail-mednli-stsb, MedEmbed-large-v0.1, and pubmedbert-base-embeddings). RAG: retrieval-augmented generation

In knowledge-intensive domains such as clinical anesthesiology, it is essential that language models demonstrate strict adherence to instructions, ensure factual grounding in retrieved data, and maintain consistency and explainability. To optimize for these requirements, we implemented a grid search strategy across four parameters: top-probability (top-p), temperature (temp), retrieval depth (top-k*)*, and the embedding model (Figure 3).

The combination of top-p (nucleus sampling) and temp (temperature) regulates the stochasticity and “creativity” of the generation model by reshaping the output logit distribution [22,23]. As illustrated in the heatmap in Figure 3, we stratified the parameter space into three stability zones: normal, fine, and very fine, which correspond to high, medium, and low levels of output variability, respectively. This stratification enables assessment of model reliability across varying degrees of deterministic constraint.

The retrieval component was tuned using the top-k parameter, which dictates the number of relevant document chunks retrieved and ranked by similarity. We evaluated three retrieval depths (top-k=4, 8, and 12) to analyze the trade-off between maximizing information recall and minimizing context noise. Additionally, four embedding models were benchmarked: a general-purpose text embedding (GTE) model (Stella_en_400M_v5) and 3 domain-specific models fine-tuned on clinical and biomedical corpora: BioBERT-mnli-snli-scinli-scitail-mednli-stsb, MedEmbed-large-v0.1, and pubmedbert-base-embeddings.

Meta-Llama-3-8B-Instruct was selected as the generation model for all RAG configurations. This model was chosen for its optimal balance of performance and efficiency, featuring a compact size of 8 billion parameters, support for prequantization, and state-of-the-art reasoning capabilities across diverse benchmarks [24].

To identify the optimal configuration, we used the BASIC exam sample questions, a set of practice items designed for the American Board of Anesthesiology examination, as the ground-truth benchmark [25]. The original dataset comprises 47 questions; however, question 25 was excluded due to its reliance on visual data interpretation, which is unsupported by the text-only structure of the Llama-3 models. Consequently, the final evaluation was performed on 46 validated items. (Table S1 in Multimedia Appendix 1) Because hyperparameter tuning was conducted on a relatively small 46-question subset, the selected parameter combinations should be interpreted as reference settings for the present experimental framework rather than universally optimal values. For the retrieval component, the textbook *Miller’s Anesthesia* [26] served as the external knowledge source, processed via a PDF parsing pipeline to facilitate vector indexing.

### Experiment 2: Large-Scale Benchmark Evaluation of Embedding-and-Retrieval-Depth-Evaluation

To assess the external validity of the RAG configurations on a comprehensive dataset, we assembled a 350-item evaluation set from *Anesthesiology Examination and Board Review (7th ed*) [19]. (Table S2 in Multimedia Appendix 1) The question pool was primarily derived from summary chapters 10 and 20, selected for their breadth and topical heterogeneity, and supplemented with items from other chapters to yield a balanced distribution. (Table 1)

**Table 1.** Distribution of the 350-item evaluation set derived from Anesthesiology Examination and Board Review (7th ed) used for large-scale benchmarking of retrieval-augmented large language models.

Chapter	Topic	Total questions (n=350)	Question indices^a^
10	Practice test	132	1‐31, 35‐62, 65‐127, 130‐135, 138‐141
17	Anesthesia for miscellaneous procedures	10	397‐399, 404, 405, 410, 413‐414; 423‐424
18	Critical care medicine	5	457‐460, 462
19	Acute and chronic pain	70	531‐600
20	Practice test	133	1‐7, 9‐60, 63‐67, 69‐89, 92‐139

a Gaps in question indices (eg, questions requiring visual or tabular interpretation) were excluded from the dataset.

The prompt templates originally depicted in Figure 2 were adapted to support a uniform 5-option format (A–E), replacing the 3-option format (A–C) used in the preliminary 46-item question set. To maintain structural consistency across the dataset, questions originally containing only four options (A–D) were normalized by appending a null placeholder (“E. Not a valid option”). This ensured that the prompt structure remained invariant for all 350 items.

To facilitate direct comparison with Experiment 1, the core architecture was preserved, using the Meta-Llama-3-8B-Instruct generation model and the same retrieval corpus (Miller’s Anesthesia).

### Experiment 3: Evaluation on the Self-RAG Pipeline

To assess the efficacy of adaptive retrieval strategies, we implemented a modified self-reflective RAG (self-RAG) architecture. This approach applies a single-generation model to answer questions and perform self-critique [27]. For the purpose of controlled comparison, we simplified the setup by removing web-search and postgeneration critique modules, focusing solely on internal knowledge retrieval.

**Figure 4.**
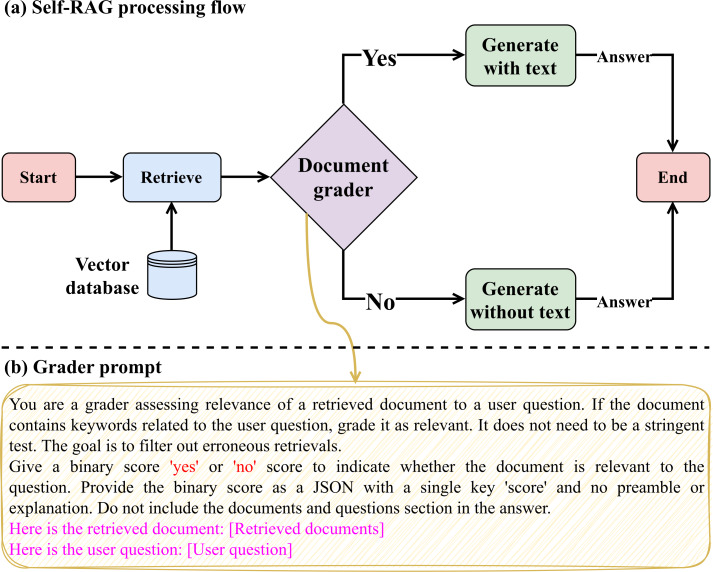
Architecture of the simplified self-reflective retrieval-augmented generation (self-RAG) pipeline. (A) The inference workflow incorporates a document grader node that evaluates the semantic relevance of retrieved chunks. Based on the binary decision ('Yes’ or ’No’), the system dynamically routes the query to either a context-augmented generation path or a context-free baseline path. (B) The prompt template used for the Document Grader, instructing the model to assess relevance and output a structured JSON response without external web search. RAG: retrieval-augmented generation.

The workflow, illustrated in Figure 4A, uses a single LLM (Meta-Llama-3-8B) to function as both the relevance evaluator and the final answer generator. Consistent with previous experiments, the stella-en_400M_v5 embedding model is used for vector retrieval.

Following the initial retrieval, a document grader module evaluates the semantic alignment between the user query and the retrieved document chunks. As detailed in the prompt template in Figure 4B, the grader is instructed to assign a binary relevance score (“Yes” or “No”) via a JSON output. This decision node dictates the generation strategy: if the retrieved content is deemed relevant (“Yes”), the system executes the “Generate with Context” protocol (standard RAG). Conversely, if the retrieved content is classified as irrelevant (“No”), the system triggers a fallback mechanism, executing “Generate without Context” (Baseline), thereby relying exclusively on the model’s parametric knowledge to prevent hallucination induced by irrelevant context.

### Experiment 4: Investigation of Retrieval Dynamics and Model Scaling

This section details a granular analysis of the RAG pipeline’s retrieval components to evaluate their impact on system performance and enhance explainability. To facilitate a controlled assessment, a representative subset of 10 questions is selected from the evaluation question set. These 10 questions were selected from the 350-question set (Table 1), where these specific questions were answered incorrectly (WRONG) across all or most settings (eg, question 139 is answered incorrectly across all tested RAG configurations) for deep retrieval analysis. The detailed locations for each question are presented in Table 2 below.

**Table 2.** Selection of the small 10-question set for deep retrieval analysis.

Question	Chapter	Topics	Question indices (in 350-question set)
1	20	Practice test	120
2	20		121
3	20		122
4	20		123
5	20		124
6	20		138
7	20		139
8	18	Critical care medicine	457
9	18		458
10	20	Practice test	47

Given the high complexity of the primary corpus (*Miller’s Anesthesia*), we established a controlled environment to isolate the generation model’s reasoning capabilities from retrieval noise. The corresponding answers and explanations from the *Anesthesiology Examination and Board Review* served as a new retrieval source, categorized into two experimental conditions:

1. Direct (oracle context): the correct explanation is injected directly into the prompt’s context field. This serves as an upper-bound baseline, providing the model with explicit ground truth.
2. Combined (distractor analysis): all ten explanations are aggregated into a single text block. The system must use the embedding model to correctly retrieve the specific segment relevant to the query from this aggregated source, simulating a “needle-in-a-haystack” retrieval task.

**Figure 5.**
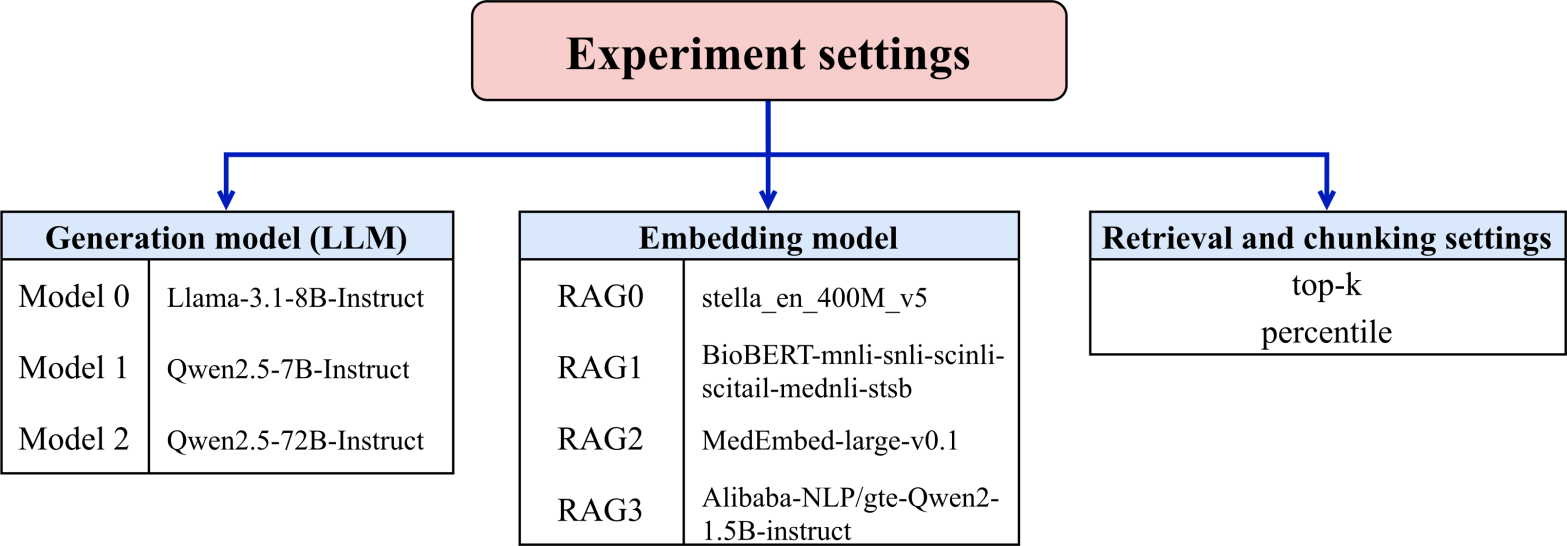
Configuration of retrieval and scaling experiments. The setup evaluates three dimensions: (Left) Generation model scaling, ranging from 8B to 72B parameters. (Center) Embedding model complexity, including general and clinical-specific encoders. (Right) Text processing strategies, comparing fixed-size recursive chunking against dynamic semantic chunking protocols. RAG: retrieval-augmented generation.

Concurrently, we examined the impact of model scaling on performance. As illustrated in Figure 5, the experimental setup included large embedding models (scaling up to 1.5 billion parameters) and generation models (up to 72 billion parameters) to test the hypothesis that increased parameter count correlates with improved accuracy. For these experiments, generation hyperparameters were fixed at the optimal values identified from the result of experiment 1 (temperature=0.1; top-p=0.1).

Finally, we evaluated the influence of text segmentation on retrieval quality. Beyond varying the retrieval depth (top-k), we compared two distinct chunking paradigms:

1. Naive recursive chunking: a mechanical approach that iteratively splits text using fixed character limits and overlap windows.
2. Semantic chunking: an advanced strategy designed to preserve semantic coherence. This method aggregates sentences based on cosine similarity scores and dynamic splitting thresholds defined by statistical measures (percentile, SD, or IQR) or gradient analysis. The latter specifically identifies boundaries by monitoring the rate of change in semantic dissimilarity between consecutive sentences. This approach mitigates the risk of fragmenting contextually related information [28,29]. In our study, percentile was selected as the threshold metric for its simplicity and practicality in our implementation in terms of graphics processing unit (GPU) video random access memory.

### Experiment 5: Evaluation of Reasoning Models Under Retrieval Complexity Constraints

Building upon the optimal configurations identified in Experiments 1‐4, this phase integrates the most effective RAG components to benchmark the next generation of reasoning-oriented LLMs. The pipeline uses the high-capacity embedding model, gte-Qwen2-1.5B-instruct, and uses semantic chunking (threshold set at the 95th percentile) to maximize context coherence.

**Figure 6.**
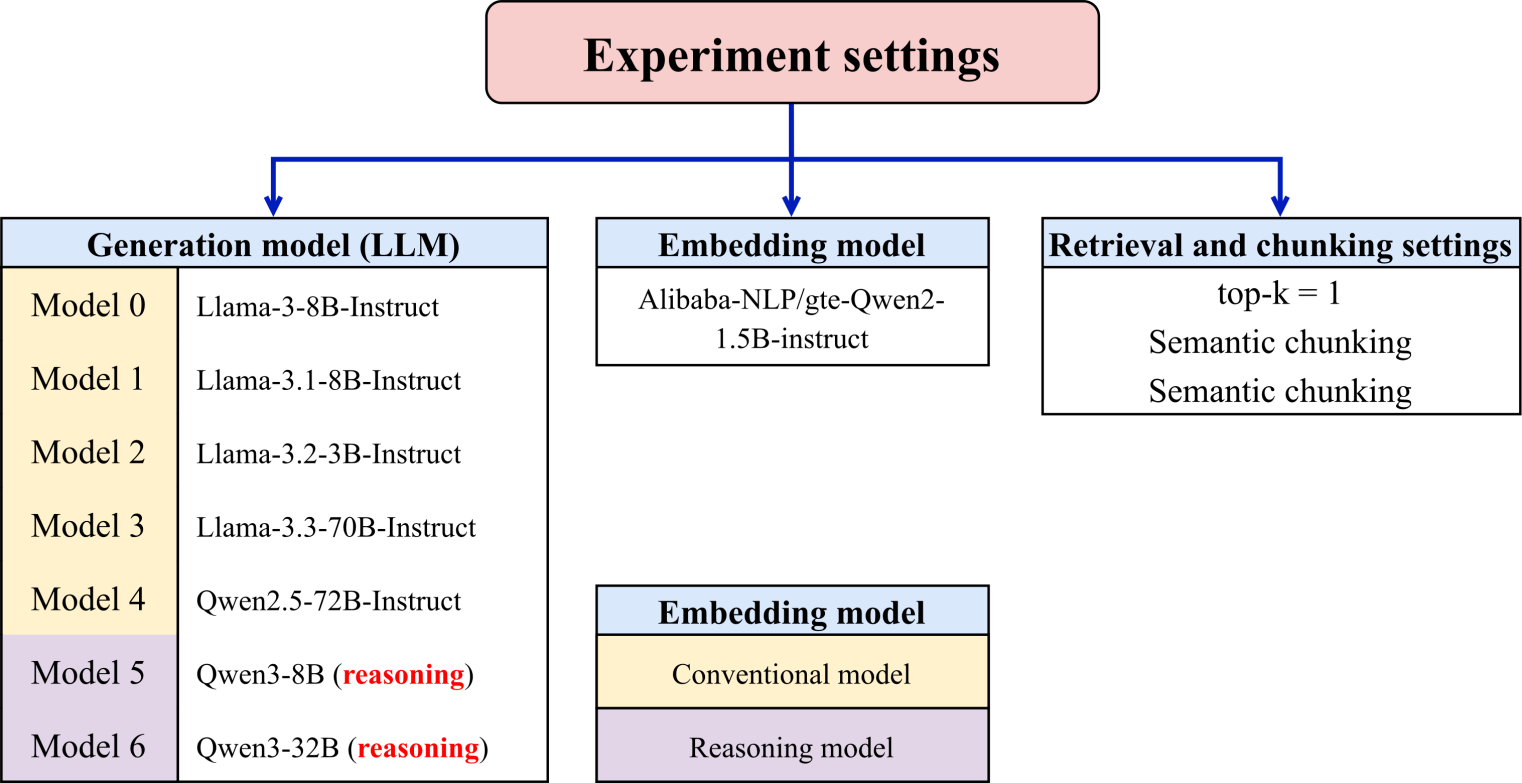
Experimental setup for benchmarking reasoning capabilities. The evaluation compares conventional instruction-tuned large language models (Yellow zone) against reasoning-enhanced models (Purple zone). Experiments use a high-capacity embedding model (gte-Qwen2-1.5B-instruct) and semantic chunking. Retrieval complexity is controlled by varying the density of irrelevant distractor text injected into the context window. LLM: large language model.

As illustrated in Figure 6, the study evaluates two distinct model categories. Conventional instruction-tuned models include the Llama series (Llama-3 to 3.3) and Qwen2.5-72B. Reasoning-enhanced models include the Qwen3 series (8B and 32B), which incorporate advanced chain-of-thought capabilities.

To ensure statistical robustness, evaluation was conducted on three randomly sampled subsets of 100 questions each, drawn from the 350-item corpus. (Table S3 in Multimedia Appendix 1) Unique random seeds were applied to ensure reproducible, independently generated sampling trials; because each 100-question subset was sampled from the same 350-item corpus, some questions could appear in more than one subset, and results are reported as the mean accuracy across the three trials. The combined 300-question analysis was used to provide more stable model-level comparisons across related retrieval-complexity conditions rather than to imply a single homogeneous statistical population.

To rigorously assess model robustness against retrieval noise, we designed a 6-level complexity spectrum. The “Bare” condition represents answers solely based on intrinsic parametric knowledge, without external context. The “Direct” condition injects the specific ground-truth explanation directly into the prompt. The “Combined” (needle-in-a-haystack) condition embeds the ground truth within a consolidated block of relevant clinical explanations. Because multiple questions in the 350-item corpus share the same source explanation block (eg, multipart vignette items that reference a common explanatory passage), the consolidated source corpus contains 263 unique explanations rather than 350. To simulate varying degrees of retrieval difficulty, the combined source is fused with increasing amounts of irrelevant distractor content (from unrelated ASA questions). This creates three graded difficulty tiers: easy, medium, and hard. With the above base combined set contains 263 explanations; we then inject 50, 125, and 274 additional (irrelevant) explanations, yielding totals of 313, 388, and 537 explanations for easy, medium, and hard, respectively. This stratification enables a granular analysis of how reasoning models distinguish relevant signals from clinical noise compared to conventional architectures.

### Statistical Analysis (Experiment 2 and 5)

The primary outcome measure was the accuracy rate, defined as the percentage of correctly answered questions. Evaluation results are presented as the mean across the randomized test subsets. To analyze performance differences across multiple model configurations, the Cochran Q test was first used to detect any overall statistically significant differences among the classifiers on the same dataset. Subsequently, the McNemar test was used for post-hoc pairwise comparisons to identify specific performance improvements between model pairs. To account for multiple hypothesis testing, a Bonferroni correction was applied to the significance threshold. All analyses were performed using Python (scipy.stats library), and a 2-tailed *P* value of <.05 was considered statistically significant.

This statistical analysis was conducted on the results obtained from experiments 2 and 5. In experiment 2, we analyzed performance differences among RAG configurations that varied in embedding models, while holding the hyperparameters (top-k, top-p, and temperature) constant. For instance, Cochran Q and McNemar tests were applied to compare the answer patterns of 350 questions between the Baseline model and the RAG0 configuration, with the hyperparameters set to top-k=4, top-p=0.1, and temperature=0.1. In contrast, Experiment 5 involved three distinct 100-question test sets for each RAG configuration, each corresponding to a different LLM model. To enhance the statistical power, the answers from these three test sets were aggregated into a single array of 300 answers (wrong or correct), enabling a more robust statistical analysis. For each analysis (eg, comparing different embedding models under the same RAG hyperparameters), we report the post-hoc pairwise McNemar results, including each configuration’s win count, Bonferroni-corrected α, and the computed *P* value.

### Computational Environment and Implementation

All retrieval and generation experiments were conducted in a Rocky Linux 8.4 environment using Python (version 3.6.8). The implementation of the RAG pipeline and model inference used a combination of the HuggingFace, Transformer, and LangChain libraries, along with the PyTorch framework. The workstation server was equipped with eight V100 GPUs (32 GB each) and 767 GB of RAM. Models with fewer than 32 billion parameters were executed on a single GPU, whereas larger models, such as the Qwen2.5-72B, required up to 4 V100 GPUs to run both the LLM and the embedding model concurrently.

## Results

### Experiment 1: Searching for an Optimal Creativity Control

**Figure 7.**
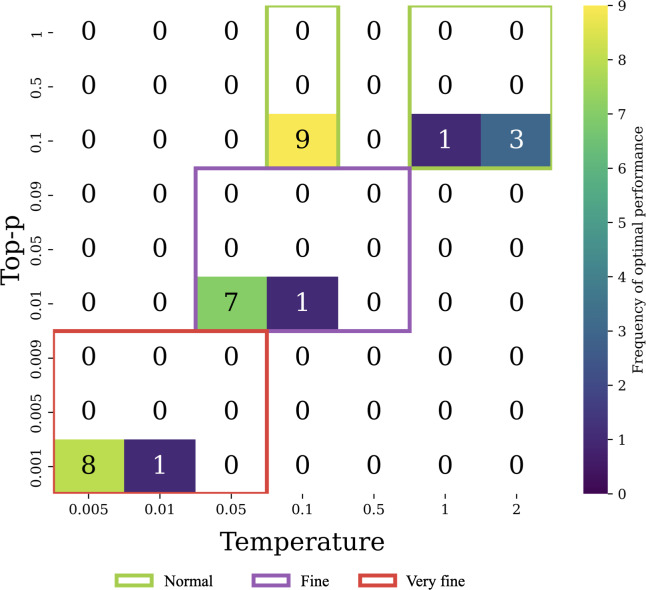
Frequency distribution of top-performing temperature and top-p configurations across the 46-question anesthesiology board-style benchmark subset used for hyperparameter optimization. The heatmap displays the number of times a specific combination of temperature (x-axis) and top-p (y-axis) yielded the highest correct ratio across all tested embedding models and retrieval depths (top-k). The color gradient and annotated cell values represent the total count of these maximum performance occurrences. Because multiple hyperparameter pairs could tie for the highest accuracy within a single experimental grid, the summed frequency across all cells was 30 rather than 15, the number of experimental grids evaluated.

To provide an overview of the hyperparameter optimization results, we first summarize the distribution of top-performing temperature and top-p configurations across all experimental settings (Figure 7). Overall, the RAG-empowered framework consistently achieved higher accuracy, as indicated by the brighter colors in Multimedia Appendix 2. Across the 15 evaluated configurations, 30 maximum-performance occurrences were identified due to ties in peak accuracy; among these, 9 were observed at the (temperature, top-p) pair (0.1, 0.1) in the NORMAL zone. This finding is supported by the frequency distribution of optimal hyperparameter configurations (temperature and top-p) across different embedding models and *top-k* values (Figure 7). In general, peak performance aligned with the most conservative pair within each region: (0.1, 0.1), (0.05, 0.01), and (0.005, 0.001) for the normal, fine, and very fine zones, respectively. The (0.1, 0.1) pair achieves the maximum accuracy 9 times, which is significantly higher than any other configuration. Additionally, this pair achieves the highest overall accuracy of 71.74%, using the MedEmbed embedding model with a top-k of 8. Based on these comprehensive results, the 3 temperature and top-p pairs, (0.1, 0.1), (0.05, 0.01), and (0.005, 0.001), were selected for subsequent experiments.

### Experiment 2: Large-Scale Benchmark Evaluation of Embedding-and-Retrieval-Depth-Evaluation

**Figure 8.**
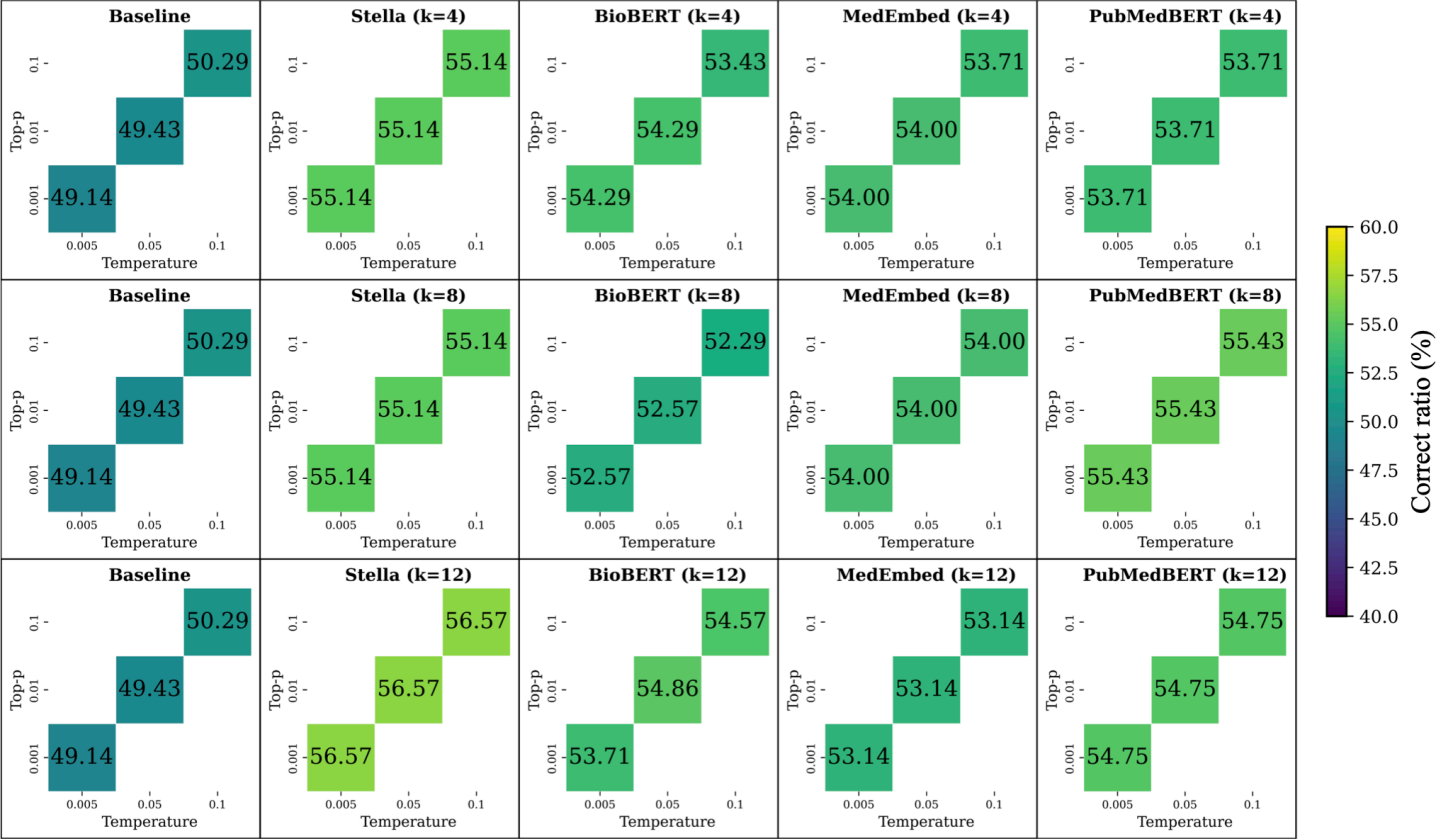
Accuracy of retrieval-augmented generation (RAG) configurations across the 350-question anesthesiology board-style evaluation benchmark. The heatmap matrix displays the performance of the three selected deterministic hyperparameter pairs (temperature and top-p) across different embedding models (columns) and retrieval depths (top-k; rows) using Meta-Llama-3-8B-Instruct as the generation model. The color gradient indicates the system's accuracy, expressed as the percentage of correct responses (Correct Ratio %). The Baseline model does not use retrieval; therefore, its performance remains constant across different top-k columns.

In contrast to the strong performance observed on the 46-question set, the larger, more topic-diverse set yields markedly different results (Figure 8). The baseline (no-RAG) model achieved 50.29%, representing a decrease of 6.21 percentage points compared to its performance on the 46-question set (Table S1 in Multimedia Appendix 1). Furthermore, the performance gains achieved via RAG were modest on this corpus: the best configuration (stella-en_400M_v5 with top-k=12 across all three pairs of temperature and top-p) yielded a maximum improvement of 6.28 percentage points over the baseline, while other settings delivered marginal improvements ranging from 2 to less than 5 percentage points.

Although Experiment 1 indicated that a medical-tuned embedding (eg, MedEmbed-large-v0.1, top-k=12) could outperform a general-text embedding, Stella_en_400M_v5*,* on a narrower set (71.7% vs 65.2% for top-p=0.1, temperature=0.1, and top-k=8; Multimedia Appendix 2), this advantage did not transfer to the larger 350-question benchmark. Embeddings trained on published biomedical corpora or clinical-note–style data may not be explicitly aligned with the specific nuances of anesthesiology question-answering, resulting in relative underperformance (Figure 8), which is consistent with a domain and topic mismatch.

Cochran Q and post-hoc McNemar test results for Experiment 2 under retrieval depths top-k=4, 8, and 12 are demonstrated in Table S4 in Multimedia Appendix 1. For the most conservative retrieval setting (top-k=4) combined with the empirically stable sampling regime (temperature=0.1*,* top-p=0.1), Cochran Q was not significant (*P*=.06). This indicates that varying the embedding model does not produce a statistically detectable change in the 350-question answer pattern. By contrast, all other Cochran Q tests reached statistical significance.

After Bonferroni correction (corrected *α*=.005), most pairwise McNemar comparisons between Baseline and individual RAG configurations were not significant. (Table S4 in Multimedia Appendix 1) This indicates that conventional RAG does not consistently induce a statistically reliable shift relative to baseline (ie, unaugmented) generation on this heterogeneous 350-item set. Nevertheless, among the tested embedders, Stella exhibits the most pronounced divergence from the baseline in several settings (demonstrating larger discordant “win” counts and comparatively smaller McNemar *P* values), which is coherent with its generally higher observed accuracy across configurations. Specifically, at a retrieval depth of top-k=12, the performance of the Stella embedding model diverged significantly from the Baseline under the two more deterministic sampling regimes (*P* value<.01 and *P* value<.01, for temp=0.05 with top-p=0.01 and temp=0.005 with top-p=0.001, respectively), surviving the Bonferroni correction.

### Experiment 3: Evaluation on the Self-RAG Pipeline

**Figure 9.**
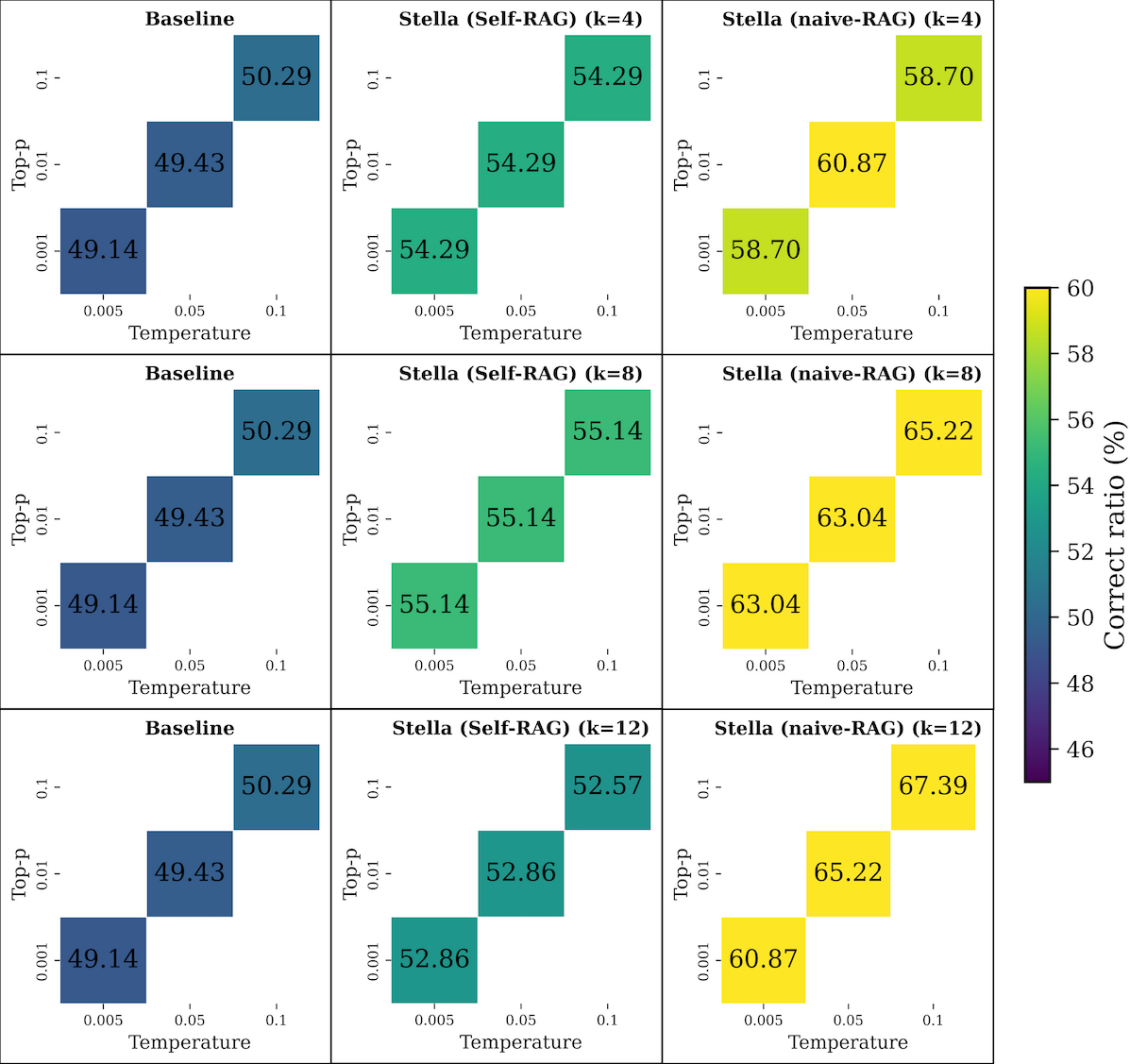
Accuracy of the self-retrieval-augmented generation (RAG) pipeline across the 350-question anesthesiology board-style benchmark under varying retrieval depths and deterministic sampling configurations.

The performance of the Self-RAG pipeline compared with the baseline model is illustrated in Figure 9. As shown in Figure 9, the self-RAG implementation yielded only marginal improvements ranging from 2.28 to 4.85 percentage points, relative to the best baseline accuracy of 50.29%. This indicates no substantial benefit over the conventional RAG system (Figure 2B).

### Experiment 4: Investigation of Retrieval Dynamics and Model Scaling

#### Performance Across Different Configurations

Table 3 reports the accuracy for each configuration under direct and combined conditions. In the direct condition, Qwen2.5-72B-Instruct had higher accuracy than Llama-3.1-8B-Instruct and Qwen2.5-7B-Instruct. In the combined condition, pairing a large LLM with a high-capacity embedding model yielded the highest accuracy. Specifically, Qwen2.5-72B-Instruct attained an 80% accuracy rate with Stella (top-k=4), MedEmbed (top-k=4), and GTE-Qwen2 (top-k=1 and 2). Furthermore, its generated answers were identical to those of the direct condition (Table 4), demonstrating both effective retrieval and stable reasoning capabilities on this subset. By contrast, smaller LLMs and lower-capacity embeddings exhibited lower accuracy in the combined condition across the same settings. Specifically, Qwen2.5-7B-Instruct achieved an accuracy of 60% with Stella when top-k=4, 60% with MedEmbed when top-k=2, and 60% with GTE-Qwen2 when top-k=2. In this diagnostic experiment, large-scale LLMs demonstrated a substantial advantage in overall accuracy compared with smaller architectures.

**Table 3.** Accuracy of generation models on the 10-question controlled retrieval subset. Performance is compared between direct ground-truth injection (Direct) and retrieval from a consolidated distractor source (combined). Results are broken down by retrieval depth (top-k) and embedding model configuration. Direct: The correct explanation was provided directly in the prompt. Combined: The model retrieved context from a single text string containing 10 distinct explanations. Stella: stella_en_400M_v5; BioBERT: BioBERT; MedEmbed: MedEmbed-large-v0.1; GTE-Qwen2: gte-Qwen2-1.5B-instruct.

Generation model	Direct	Combined				
		top-k	Stella	BioBERT	MedEmbed	GTE-Qwen2
Qwen2.5-7B-Instruct	60%	1	50%	30%	40%	60%
		2	60%	50%	60%	60%
		4	60%	40%	50%	50%
Llama-3.1-8B-Instruct	70%	1	60%	40%	30%	60%
		2	30%	30%	30%	60%
		4	50%	60%	50%	60%
Qwen2.5-72B-Instruct	80%	1	70%	50%	60%	80%
		2	70%	50%	70%	80%
		4	80%	70%	80%	70%

**Table 4.** Item-level response comparison on the 10-question evaluation subset. The table maps the specific multiple-choice answers generated by the Qwen2.5-72B-Instruct model under the Direct (oracle context) and Combined (retrieval-augmented) conditions, both using the gte-Qwen2-1.5B-instruct embedding model, against the ground truth. The identical response patterns between the two experimental conditions demonstrate the model’s stable reasoning capabilities despite the introduction of distractor text in the Combined setting.

Question	1	2	3	4	5	6	7	8	9	10
Ground truth	E	D	E	B	D	C	B	C	D	E
Direct	E	D	D	B	D	C	B	C	D	A
Combined	E	D	D	B	D	C	B	C	D	A

#### Retrieval Inspection

To evaluate retrieval precision, we assessed top-1 retrieval accuracy on the 10-item diagnostic set (top-k=1) using a medical-tuned embedder (BioBERT) and a high-capacity general-text embedder (GTE-Qwen2). As presented in Table 5, the bracketed indices indicate the specific explanation passage retrieved from the Anesthesiology Examination and Board Review for each question. GTE-Qwen2 achieved a 90% (9 out of 10) match rate, with a single error in which it retrieved the first question’s explanation for the second question. In contrast, BioBERT retrieved only 40% (4 out of 10) of the correct documents. Consequently, we adopted GTE-Qwen2 as the default embedding model for subsequent comparisons between conventional and reasoning-oriented LLM pipelines.

**Table 5.** Top-1 document retrieval accuracy on the 10-question evaluation subset. The table displays the specific document indices retrieved by the BioBERT (RAG 1) and GTE-Qwen2 (RAG 3) embedding models at a retrieval depth of top-k=1. Bracketed numbers indicate the index of the retrieved explanation passage.

Questions	1	2	3	4	5	6	7	8	9	10
Ground truth	[1]	[2]	[3]	[4]	[5]	[6]	[7]	[8]	[9]	[10]
BioBERT	[9]	[1]	[3]	[7]	[5]	[6]	[6]	[8]	[1]	[9]
GTE-Qwen2	[1]	[1]	[3]	[4]	[5]	[6]	[7]	[8]	[9]	[10]

#### Chunking Strategies and Semantic Chunking

**Figure 10.**
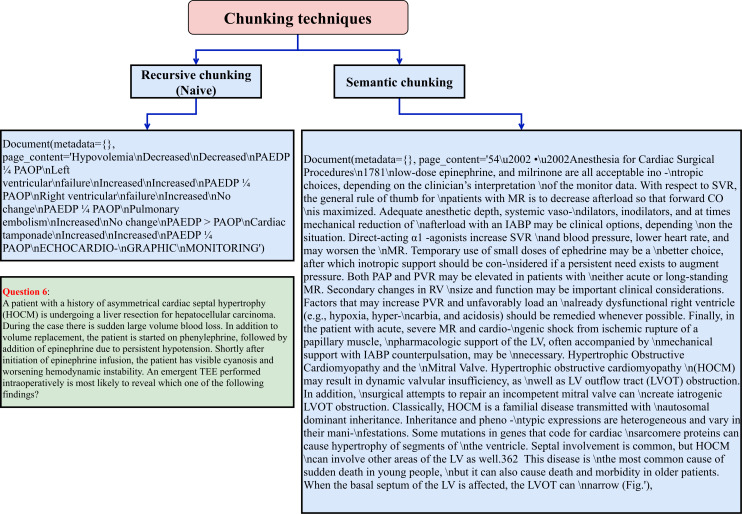
Qualitative comparison of retrieved document context between naive recursive and semantic chunking strategies. The figure illustrates the raw text chunks retrieved for Question 6 of the diagnostic subset. (Table 2) The naive recursive approach (left) yields a fragmented, artifact-laden text segment containing clipped headers and layout markers, whereas semantic chunking (right) successfully extracts a contextually coherent and relevant paragraph from Miller’s Anesthesia.

We compared naïve recursive chunking (fixed chunk size and overlap) with semantic chunking (context-aware segmentation) on the *Miller’s Anesthesia* textbook, which is highly formatted and prone to page-layout artifacts [26]. As demonstrated by a representative question (Figure 10), naïve recursive chunking produced fragmented out-of-context segments (eg, clipped phrases and header remnants) that propagate into the vector store and prompts, thereby diluting retrieval precision. In contrast, semantic chunking yielded a contextually coherent paragraph (page 1781) that is topically aligned with and adjacent to the gold-standard evidence for Question 6 (page 1782). Although primarily illustrative, this example highlights the expected advantages of preserving discourse boundaries in complex medical texts: fewer spurious tokens, greater passage relevance, and cleaner context injection. Accordingly, we adopted semantic chunking as the default strategy in subsequent experiments and evaluated its broader impact in the following sections.

To further quantify retrieval quality, we performed a focused retrieval relevance comparison using the 10-question diagnostic retrieval subset. (Table 2) Under identical retrieval settings, semantic chunking achieved successful retrieval in all 10 questions (10/10, 100%), whereas recursive chunking achieved successful retrieval in 8 of 10 questions (8/10, 80%). In addition, semantic chunking consistently retrieved more contextually complete passages with higher overlap to the reference explanations, whereas recursive chunking frequently produced fragmented or only partially overlapping text segments. (Table S5 in Multimedia Appendix 1).

### Experiment 5: Evaluation of Reasoning Models Under Retrieval Complexity Constraints

**Figure 11.**
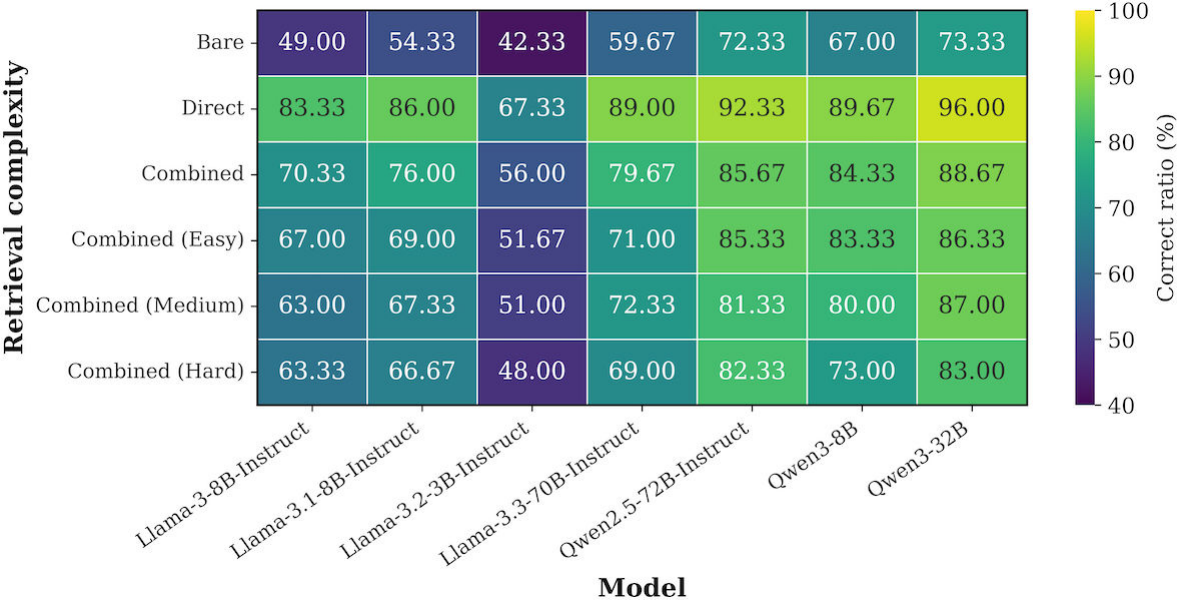
Accuracy of conventional and reasoning-oriented large language models across progressively complex retrieval conditions in the anesthesiology board-style benchmark. Retrieval complexity increased from direct oracle-context injection to distractor-heavy retrieval settings (easy, medium, and hard), enabling evaluation of robustness to noisy contextual inputs. The matrix compares the performance (Correct Ratio %) of seven large language models across progressively difficult context retrieval conditions. The models evaluated include both conventional architecture and reasoning-oriented models. Model 0 = Llama-3-8B-Instruct; Model 1 = Llama-3.1-8B-Instruct; Model 2 = Llama-3.2-3B-Instruct; Model 3 = Llama-3.3-70B-Instruct; Model 4 = Qwen2.5-72B-Instruct; Model 5 = Qwen3-8B; Model 6 = Qwen3-32B.

Figure 11 reports the mean accuracy across three 100-item sets for 7 LLMs as retrieval complexity increases. As demonstrated in the figure, accuracy consistently declined as the retrieved evidence became longer and contextually denser, progressing from the direct to the combined (hard) conditions.

The reasoning-oriented Qwen3-32B model achieved the highest accuracy, exceeding the largest tested conventional model, Qwen2.5-72B-Instruct by 1‐6 percentage points across conditions (eg, increasing from 92% to 96% in the Direct, from 86% to 89% in the combined, and from 81% to 87% in the Combined Medium setting). Following Qwen2.5-72B-Instruct, the Qwen3-8B model also demonstrated strong performance, achieving 67% in the Bare, 90% in the direct, and 84% in the combined setting, higher than all models in the Llama family. Within the Llama family, while the Llama-3.3-70B model outperformed the smaller Llama-3-8B-Instruct*,* Llama-3.1-8B-Instruct, and Llama-3.2-3B-Instruct models across all conditions (eg 80% vs 70%, 76%, and 56% in the combined condition; 69% vs 63% and 67%, and 48% in the hard condition), the gains were diminished when the retrieval difficulty increased, especially against Llama-3.1-8B-Instruct variant.

**Figure 12.**
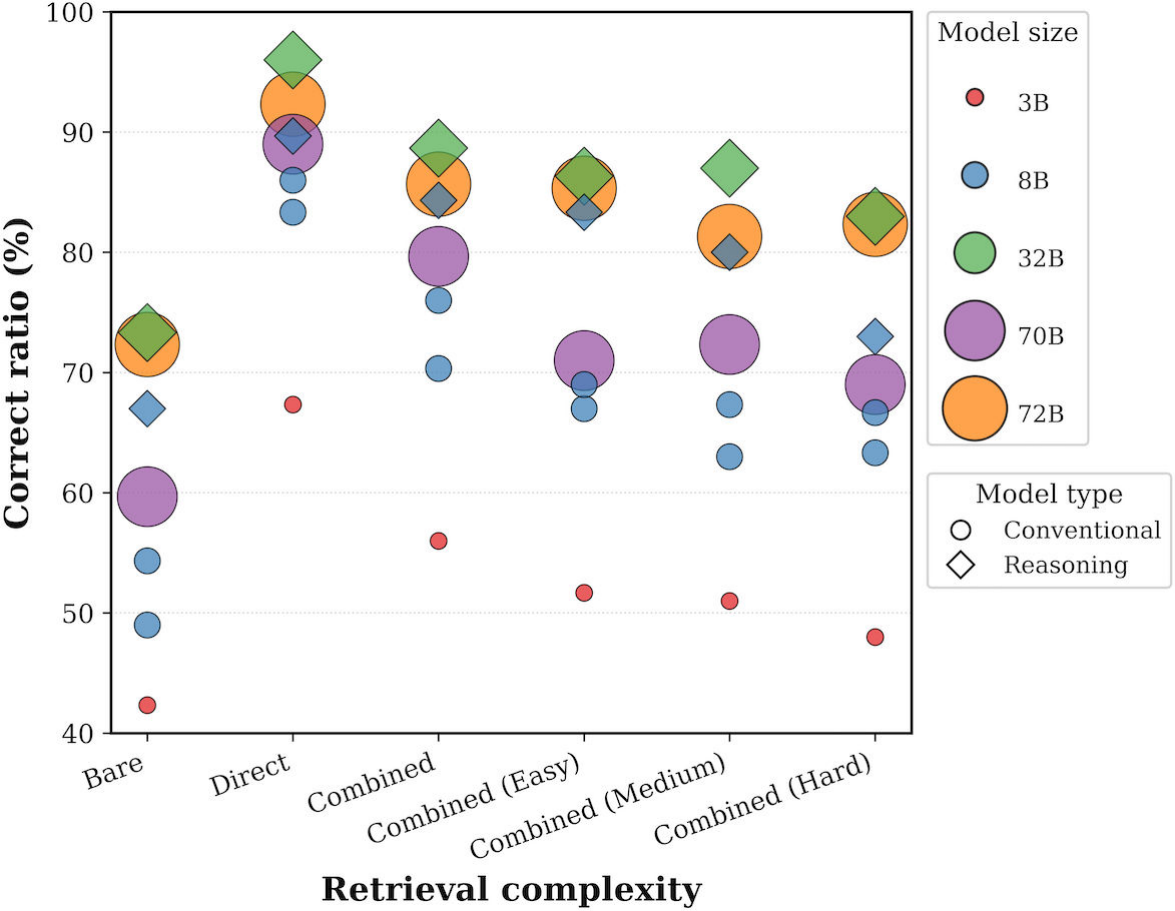
Relationship between model size, reasoning-oriented architecture, and accuracy across varying retrieval-complexity conditions in the anesthesiology board-style benchmark. The figure illustrates the correct ratio (%) for models of different sizes and types, including conventional and reasoning models. The 8B size appears twice within each retrieval-complexity condition because both Llama-3-8B-Instruct and Llama-3.1-8B-Instruct have 8 billion parameters.

Figure 12 shows the relationship between model scale, reasoning capability, and accuracy across all retrieval conditions. Across the evaluated settings, larger models generally achieved higher correct ratios than smaller models within the same model family (ie, Llama or Qwen). This performance gap by model size became more pronounced as retrieval complexity increased. In addition, reasoning-enabled models maintained accuracy levels comparable to those of larger conventional models and exhibited a more stable accuracy across different retrieval conditions.

Experiment 5’s Cochran Q and post hoc McNemar comparisons across the 7 LLM-based RAG systems at multiple retrieval-complexity tiers are reported in Table S6 in Multimedia Appendix 1. Cochran Q was highly significant across all tiers (*P*<.05). Pairwise McNemar results demonstrated that the Qwen family, especially the reasoning models (Qwen3-8B and Qwen3-32B), exhibited the most consistent and substantial divergence from the Llama series. Specifically, Qwen3-32B significantly outperformed 4 nonreasoning models (Llama-3-8B-Instruct, Llama-3.1-8B-Instruct, Llama-3.2-3B-Instruct, and Llama-3.3-70B-Instruct) in bare, combined, and easy conditions, 5 models in direct, medium, and hard conditions (Llama-3-8B-Instruct, Llama-3.1-8B-Instruct, Llama-3.2-3B-Instruct, Llama-3.3-70B-Instruct, and Qwen3-8B), but nonsignificantly outperformed nonreasoning Qwen2.5-72B-Instruct model.

## Discussion

### Principal Findings

We compared a RAG pipeline with an unaugmented baseline LLM and evaluated variations in core components (generator scale, embedding model, chunking strategy, sampling hyperparameters, and reasoning capability). On our benchmarks, the RAG framework delivered higher and more consistent accuracy than baseline generation when sampling temperature and top-p were tuned for stability; among the tested low-stochasticity configurations, temperature=0.1 and top-p=0.1 frequently yielded comparatively stable performance across experiments, although the exact optimal configuration varied across retrieval settings and embedding models. (Multimedia Appendix 2 and Figure 7) These findings suggest that lower-stochasticity sampling settings may improve performance stability in structured medical question-answering benchmarks, as excessively high parameter values can introduce variability that compromises factual consistency, whereas overly restrictive settings may reduce necessary inferential flexibility.

Retrieval quality emerged as the primary determinant of overall performance. As retrieval depth increased, configuration-dependent differences in model reasoning were amplified, highlighting the delicate balance between information recall and context noise. Although medical-tuned embedders demonstrated strong performance on narrow topical subsets, their advantage did not transfer to the broader 350-item corpus, a finding that is consistent with a domain and task mismatch. In contrast, a high-capacity GTE model (gte-Qwen2-1.5B-instruct) achieved higher retrieval precision and improved end-to-end accuracy. Table S4 in Multimedia Appendix 1 suggests that model capacity, combined with broad pretraining, can outweigh narrow biomedical fine-tuning when the target data distribution shifts, providing richer, more discriminative vector representations for precise knowledge extraction.

The choice of chunking strategy also critically influenced context quality. Naïve recursive chunking (fixed windows) produced fragmented, out-of-context segments from the *Miller’s Anesthesia* textbook [26], injecting headers and incomplete sentences into the vector store. Semantic chunking reduced these fragmentary chunks and retrieved passages with greater semantic alignment to the gold-standard evidence in illustrative cases. (Figure 10 and Table S5 in Multimedia Appendix 1) Consequently, we adopted it as the default processing method in subsequent experiments. Furthermore, our evaluation of a simplified Self-RAG variant demonstrated that simply increasing the architectural complexity of the pipeline does not necessarily yield clinically meaningful improvements, as the underlying generation models often inherit fundamental limitations in deep medical knowledge representation. (Figure 9) Therefore, within the present implementation, optimization of retrieval quality and contextual refinement appeared to contribute more substantially to performance than the additional postretrieval grading mechanism.

Reasoning-oriented LLMs demonstrated robust competitive performance. Among the conventional (nonreasoning) models, Qwen2.5-72B-Instruct served as the strongest baseline, achieving higher scores than its Llama model family counterparts, which is consistent with prior anesthesia benchmarks [30]. On the studied datasets, the reasoning-centric Qwen3-32B achieved even higher accuracy than the conventional Qwen2.5-72B-Instruct model. (Figures 11 and 12) These findings suggest that reasoning-oriented training and related model characteristics may improve robustness under complex retrieval settings, as increases in raw parameter capacity do not guarantee proportional performance gains on highly specialized anesthesiology examination benchmarks. Across increasing levels of retrieval difficulty, reasoning-oriented models demonstrated greater robustness to noisier and denser contextual inputs. This points to a complementary architectural approach, where a reasoning model, combined with optimized retrieval, can improve performance in structured medical question-answering benchmarks and may inform future development of medically oriented retrieval systems. However, these differences should not be interpreted as arising solely from reasoning capability, as variations in architecture, pretraining data, instruction tuning, and posttraining optimization may also contribute.

To further characterize failure modes, we reviewed all eligible cases in which Qwen3-32B answered correctly under the Direct condition but incorrectly under the Combined condition in Experiment 5. Among the 24 reviewed cases, 19 were associated with retrieval mismatch, in which the retrieved passages did not contain the key evidence needed to support the correct answer. Four additional cases reflected insufficient or only partially relevant retrieval, while 1 case reflected failure to correctly use partially relevant retrieved information despite the presence of related contextual evidence. These findings suggest that most residual errors under retrieval-complexity settings were primarily associated with retrieval quality and distractor-heavy contextual inputs rather than solely reflecting lack of underlying question-answering capability. Detailed examples and retrieved contexts are provided in Table S7 (Multimedia Appendix 1).

### Comparison With Prior Work

Initial explorations of LLMs in anesthesiology primarily evaluated the intrinsic parametric knowledge of standalone models. For instance, Shay et al [31] assessed ChatGPT-3.5 on ABA–style practice questions, reporting an overall accuracy of 56.2%, falling short of standard passing thresholds. Similarly, Angel et al [12] demonstrated that while early models like Bard (46.7%) and GPT-3 (58.3%) failed to pass the ABA written examination, the advanced closed-source GPT-4 achieved 78.3%. These findings closely mirror our baseline results; our unaugmented, open-source Llama-3-8B-Instruct, Llama-3.1-8B-Instruct, and Llama-3.2-3B models achieved comparable 49%, 54%, and 42% accuracies, respectively. (Figure 8) In addition, our larger-scale Qwen2.5-72B-Instruct also achieved a comparable 72% accuracy. (Figure 8) This comparison confirms that the raw parametric knowledge of smaller models aligns with earlier foundation models and is insufficient for complex anesthesiology examinations.

More recently, the literature has demonstrated how RAG and reasoning can elevate model accuracy on medical licensing thresholds. Recent research by Elkin et al [32] on the USMLE revealed that while large Llama-3-70B models passed the exams natively and achieved up to 92% accuracy with RAG, integrating semantic clinical knowledge was necessary to help a smaller Llama-2-13B model cross the 60% passing threshold on Step 3 (scoring 60.2%). This scale-dependent benefit closely aligns with our findings: our unaugmented Llama-3.1-8B-Instruct baseline (54%) saw only modest gains with RAG (76%), whereas our larger conventional Qwen2.5-72B-Instruct model equipped with RAG achieved 72% and 86% accuracies without and with RAG, respectively (Figure 8). Concurrently, meta-analyses of global medical exams show that advanced reasoning models dominate medical examination benchmarks, with models like GPT-o1 and DeepSeek-R1 achieving overall accuracy rates of 95.4% and 92%, respectively [33]. This emphasizes the critical role of robust test-time reasoning in specialized medical contexts [30]. Similarly, our reasoning Qwen3-32B model integrated with an optimized RAG pipeline achieved up to 89% accuracy on complex, distractor-heavy retrieval tasks (Figure 11).

### Limitations

Although this work evaluates model accuracy on curated anesthesia question sets, it does not constitute clinical validation in real-world patient care scenarios. In terms of document complexity control, the Direct context setting estimates a theoretical performance ceiling under perfect retrieval and is not deployable in practical applications. Several findings (eg, table-level retrieval metrics or single-question chunking illustrations) are based on small diagnostic subsets; broader replication is needed to confirm generalizability. Moreover, this study did not exhaustively audit failure modes (eg, hallucination typology and reasoning chain validity) or conduct clinical expert adjudication of the generated rationales. Finally, evaluations of model safety, bias, and calibration were out of scope and require a significant dedicated study before any clinical deployment can be considered. In addition, the evaluated benchmark consisted of structured text-based anesthesiology board-style questions. Accordingly, the present findings are most directly applicable to controlled medical question-answering benchmarks and may not generalize to open-ended clinical dialogue, real-time guideline retrieval, or multimodal anesthesiology tasks involving images, physiologic waveforms, or tabular data.

### Conclusions

In conclusion, our results demonstrate that a RAG-powered LLM pipeline can achieve greater than 80% accuracy on complex anesthesiology board-style examinations under controlled retrieval conditions (oracle or curated context in experiments 4 and 5), whereas under open RAG retrieval on the heterogeneous 350-question benchmark in experiment 2, gains over the unaugmented baseline were modest (50.29% to 56.57%). These findings suggest its potential utility in benchmark-oriented evaluation settings. The relatively compact size of the evaluated open-source models (eg, 8 billion to 72 billion parameters), compared with massive, proprietary foundation LLMs like GPT-4, may facilitate local deployment and real-time responses. This mitigates critical infrastructural issues such as network latency, server overload, and patient privacy concerns. Moreover, the RAG framework facilitates dynamic knowledge updates, allowing new medical guidelines to be integrated into the document corpus without requiring expensive, computationally intensive model retraining. By explicitly outputting both the generated reasoning steps and the retrieved context, the system provides partial transparency into the mechanisms driving its final answer. However, optimizing a RAG system requires significant methodological effort to tune hyperparameters and test retrieval strategies to ensure reliable, accurate results in structured benchmarking settings.

## Supplementary material

10.2196/97902 Multimedia Appendix 1 Question sets, retrieval and chunking analyses, statistical comparisons, and item-level failure analyses for benchmarking retrieval-augmented large language models on anesthesiology board-style questions.

10.2196/97902 Multimedia Appendix 2 Heatmap visualization of hyperparameter optimization results (Experiment 1). This appendix includes the heatmap visualization for the correct ratio between different RAG system configuration. The grid is organized by embedding model (columns) and retrieval depth (top-k; rows). Within each individual heatmap, the horizontal and vertical axes represent the temperature (temp) and top-p values, respectively. The color gradient indicates the model’s accuracy, expressed as the percentage of correct responses out of 46 benchmark questions. (correct ratio %).

## Abbreviations

ABA: American Board of Anesthesiology
GPU: graphics processing unit
GTE: general-text embedding
Llama: Large Language Model Meta AI
LLM: large language model
RAG: retrieval-augmented generation
USMLE: United States Medical Licensing Examination
